# Intracellular Delivery of Lipopolysaccharide Induces Effective Th1-Immune Responses Independent of IL-12

**DOI:** 10.1371/journal.pone.0068671

**Published:** 2013-07-17

**Authors:** Sachiko Watanabe, Joe Inoue

**Affiliations:** 1 Department of Biosciences, School of Science and Graduate School of Science, Kitasato University, Japan; 2 Department of Immunology and Cell Biology, Graduate School of Medicine, Kyoto University, Japan; Glaxo Smith Kline, Denmark

## Abstract

Lipopolysaccharide (LPS) is responsible for many of the inflammatory responses and pathogenic effects of Gram-negative bacteria, however, it also induces protective immune responses. LPS induces the production of inflammatory cytokines such as TNF-α, IL-6, and IL-12 from dendritic cells (DCs) and macrophages. It is thought that IL-12 is required for one of the protective immune responses induced by LPS, the T helper 1 (Th1)-immune response, which include the production of IFN-γ from Th1cells and IgG2c class switching. Here, we clearly demonstrate that intracellular delivery of LPS by LPS-formulated liposomes (LPS-liposomes) does not induce the production of inflammatory cytokines from DCs, but enhances Th1-immune responses via type-I IFNs, independent of IL-12. Collectively, our results strongly suggest that LPS-liposomes can effectively induce Th1-immune responses without inducing unnecessary inflammation, and may be useful as an immune adjuvant to induce protective immunity.

## Introduction

Lipopolysaccharide (LPS) is responsible for many of the inflammatory responses and pathogenic effects of Gram-negative bacteria. LPS recognition has been well studied, and Toll-like receptor 4 (TLR4) is the best-characterized LPS sensor [1]–[6]. TLR4 signaling is also well studied, and it is known that 2 major pathways, the MyD88-dependent and TRIF-dependent signaling pathways, are activated when TLR4 recognizes LPS [4]–[7]. The MyD88-dependent pathway is activated at the plasma membrane and induces inflammatory responses such as the production of TNF-α, IL-6, and IL-12 via activation of MAPK and NFκB in the early phase. On the other hand, the TRIF-dependent pathway is activated when LPS is taken into the cell [8], [9]. Recently, in support of this, Kagan *et al.* used endocytosis inhibitors and showed that endocytosis of TLR4 with LPS initiates the TRIF-dependent pathway in early endosomes [10]. TRIF-dependent signaling induces the production of type-I IFN, which activates anti-viral responses, and chemokines such as RANTES (also known as CCL5) via activation of IRF-3 and NFκB in the late phase [11].

IL-12 is involved in the differentiation of naive T cells into T helper 1 (Th1) cells by heat-killed bacteria [12]. It has been reported that IL-12p40^−/−^ mice are defective in IFN-γ production and Th1-immune responses by LPS [13]. MyD88^−/−^ mice also have a profound defect in Th1-immune responses by LPS [14]. Additionally, DCs from MyD88^−/−^ mice are defective in the production of IL-12 by LPS and induce IL-4–producing Th2 cells but not IFN-γ–producing Th1 cells [15]. Collectively, IL-12 production via the MyD88-dependent pathway is essential for the Th1-immune responses induced by LPS. However, the MyD88-dependent pathway also induces the production of inflammatory cytokines such as TNF-α and IL-6 from immune cells, and this cytokine production sometimes causes septic shock with cytokine storm [16]. In contrast, it has recently been reported that TRIF-biased TLR4 agonists can be used as vaccine adjuvants with low toxicity [17]. TRIF-biased TLR4 agonist is a safe adjuvant, however, its ability to induce immune responses is weaker than that of LPS. Hence, adjuvants that defective in activation of MyD88-dependent pathway will be safer, however, it is difficult to induce effective and protective immune responses, such as IFN-γ-producing Th1 cells, Th1-immune responses [15], [17].

In the present study, we newly prepared LPS-formulated liposomes (LPS-liposomes) to activate only the TRIF-dependent pathway via endocytosis [18]. In this study, we focused on the effect of LPS-liposomes in DCs which is essential for inducing adaptive immune responses. As expected, LPS-liposomes activated the TRIF-dependent pathway, but not the MyD88-dependent pathway, in DCs. IL-12 production was significantly decreased, but IFN-β production was up-regulated by LPS-liposomes. Surprisingly, LPS-liposomes enhanced Th1-immune responses compared with LPS. We also found the induction of Th1-immune responses by LPS-liposomes was depended on type-I IFNs, and independent of IL-12. These results strongly suggest that LPS-liposomes can effectively induce Th1-immune responses without inducing unnecessary inflammation, and may be useful as an immune adjuvant to induce protective immunity.

## Materials and Methods

### Mice

Female C57BL/6 mice, purchased from Japan SLC (Shizuoka, Japan), were used at 8–12 weeks of age. Wild-type C57BL/10ScSn (WT), IL-12p35-defective C57BL/10 (IL-12p35^−/−^), and IFN-α/β receptor-defective C57BL/10 (IFN-α/βR^−/−^) mice were obtained from the Max-Planck Institute for Immune biology and Epigenetics (Freiburg, Germany). Female MyD88-defective (MyD88^−/−^) and TRIF-defective (TRIF^−/−^) mice were purchased from Oriental Yeast co., ltd. (Tokyo, Japan). All mice were housed in a specific pathogen-free environment at the Kitasato University School of Science in strict accordance with the Institutional Animal Care and Use Committee (IACUC) Guidelines. This study was carried out in strict accordance with the recommendations in the Guide for the Care and Use of Laboratory Animals of Kitasato University School of Science. The protocol was approved by the Committee on the Ethics of Animal Experiments of Kitasato University School of Science (Permit Number: SA1014). All efforts were made to minimize suffering.

### Reagents

Highly purified LPS from *S. abortus equi* was kindly provided by Dr. Chris Galanos (Max-Planck Institute for Immune biology). Anti-CD11c, anti-MHC-II, anti-CD40, anti-CD80, and anti-CD86 antibodies for flow cytometry were purchased from BD Pharmingen (San Diego, CA). Anti-IRF3, anti-phospho-IRF3 (Ser396), anti-IκBα and anti-GAPDH antibodies for immunoblot analysis were purchased from Cell Signaling Technology (Tokyo, Japan). AP-conjugated rabbit anti-mouse IgG1 antibody for ELISA was purchased from Invitrogen. HRP-conjugated goat anti-mouse IgG2c antibody for ELISA was purchased from Thermo Scientific. 1,2-Dioleoyl-3-trimethylammonium-propane (DOTAP) and 1,2-dipalmitoyl- sn-glycero-3-phosphoethanolamine-N-[methoxy (polyethylene glycol)-2000] (DPPE-PEG) were obtained from Avanti Polar Lipids (Birmingham, AL). Ovalbumin (OVA) was obtained from Sigma (Tokyo, Japan).

### Generation of DCs

Bone marrow (BM)-derived DCs (BMDCs) were generated as described previously [19]. Briefly, BM cells were obtained from mice and cultured in RPMI 1640 containing 10% fetal calf serum (FCS) and 10 ng/mL murine granulocyte macrophage colony-stimulating factor (GM-CSF). Every 2 d, non-adherent cells were discarded and the remaining cells were supplied with fresh medium containing 10 ng/mL murine GM-CSF. At day 6, loosely adherent cells were harvested by gentle pipetting and cultured for a further 2 d. At day 8, the non-adherent cells were harvested.

### Preparation of Liposomes

DOTAP was dissolved in chloroform, vacuum-desiccated, and hydrated by vortexing with sterilized phosphate-buffered saline (PBS) for liposomes, and LPS (10–100 µg/mL) in sterilized PBS for LPS-liposomes, and diluted in PBS to obtain a final LPS concentration of 1 µg/mL. LPS (1 µg/mL), LPS-lipoosmes (LPS concentration of 1 µg/mL) and liposomes (same DOTAP concentration of LPS-liposomes) was diluted in the medium were used for the experiments. The size of LPS-liposome was 272.5±65.3 nm (DLS-7000, Otsukaelectronics, Japan), and ζ-potential was 8.5±1.8 (Laser Zee Model501, Pen Kem). For the encapsulation of OVA, DOTAP and DPPE-PEG were dissolved in chloroform, vacuum-desiccated, and hydrated by vortexing with a mixture of LPS (1 mg/mL) and OVA (1 mg/mL) in PBS, and diluted in PBS to obtain a final concentration of 100 µg/mL. Following hydration, the dispersion was sonicated for 1 min in a bath sonicator (Bioruptor, Cosmo Bio, Tokyo, Japan).

### Cytokine Determination

BMDCs (1.5×10^5^) from WT mice were stimulated with LPS or LPS-liposomes (100 ng/mL) for 2 h (TNF-α), 9 h (IFN-β) or 24 h (IL-6, IL-12p40, and RANTES). BMDCs (1.0×10^5^) from MyD88^−/−^ and TRIF^−/−^ mice were stimulated with LPS or LPS-liposomes (100 ng/mL) for 9 h (IFN-β) or 24 h (IL-12p40). Cytokine levels were determined by ELISA. WT mice were intravenously treated with LPS or LPS-liposomes (10 µg per mouse). At 1.5 h after treatment, sera were harvested and the level of TNF-αin the sera was determined by ELISA.

### ELISA

TNF-α, IL-6, IL-12p40, IFN-γ, and IL-4 were measured by ELISA using OptEIA mouse cytokine detection kit (BD Biosciences). IFN-β was measured by ELISA using mouse IFN-β ELISA Kit (PBL interferon source). RANTES was measured by ELISA using Quantikine mouse RANTES ELISA Kit (R&D Systems).

### RT-PCR

BMDCs (1×10^6^) from WT mice were stimulated with LPS or LPS-liposomes (10 or 100 ng/mL) for 4 h, and mRNA levels were determined by RT-PCR. Total RNA (1 µg) was isolated using Isogen solution (Nippon Gene, Toyama, Japan), as reported previously [20], [21]. cDNA was synthesized using SuperScript III. Then, cDNAs were amplified with primers specific for *IFN-β* and *β-actin*.

### Immunoblot Analysis

BMDCs (1×10^6^) from WT mice were stimulated with LPS or LPS-liposomes (100 ng/mL) for 0–120 min. Cells were lysed with ice-cold RIPA lysis buffer containing protease inhibitors, and the extracts were subjected to immunoblot analysis. Immunoblot analysis was performed using anti-IRF3, anti-phospho-IRF3 (Ser396), anti-IκBα and anti-GAPDH antibodies, visualized with HRP conjugate substrate system or an enhanced chemiluminescence detection system. Band intensity was quantified with Image J 1.45.

### Flow Cytometry

WT mice were intravenously treated with LPS or LPS-liposomes (10 µg per mouse). At 6 h after treatment, splenocytes were harvested and stained with PE-conjugated anti-CD11c, FITC-conjugated anti-MHC-II and CD80, and biotin-conjugated anti-CD40 and anti-CD86. Biotinylated mAbs were detected using streptavidin-PE-Cy5. The cells were analyzed using a flow cytometer EPICS ELITE (Beckman Coulter, CA), and WinMDI for analysis software. BMDCs (5.0×10^5^) from MyD88^−/−^ and TRIF^−/−^ mice were stimulated with LPS or LPS-liposomes (100 ng/mL) for 48 h, and stained with APC-Cy7-conjugated anti-CD11c, PE-conjugated anti-CD40, and PE-conjugated anti-CD86. The cells were analyzed using a flow cytometer FACS Canto II (BD Biosciences), and Flowjo for analysis software.

### Preparation of Mice Splenocytes

WT, IL-12p35^−/−^, and IFN-α/βR^−/−^ mice were immunized with OVA and LPS, or LPS-OVA-liposomes (10 µg each per mouse, i.v.). After 1 week, spleens were removed, and splenic single-cell suspensions were prepared [22], [23]. Splenocytes from immunized mice (5×10^5^) were incubated with OVA (1 mg/mL) for 72 h and IFN-γ and IL-4 levels were determined by ELISA. The splenocytes were incubated in RPMI-1640 medium supplemented with 10% FCS, 100 U/mL penicillin, 100 µg/mL streptomycin, 2 mM l-glutamine, and 5×10^–5^ M 2-mercaptoethanol.

### OVA-specific Antibody Determination

WT, IL-12p35^−/−^, and IFN-α/βR^−/−^ mice were immunized with OVA and LPS, or LPS-OVA-liposomes (10 µg each per mouse, i.v.) and boosted after 2weeks. After a further 2 weeks, sera were harvested and poured into an OVA-coated ELISA-plate, and incubated overnight. Following washes with PBS containing 0.05% Tween-20, the wells were treated with AP-conjugated-anti-mouse IgG1 or HRP-conjugated-anti-mouse IgG2c for 2 h. Following washes, enzyme reaction was performed using *p*-nitrophenyl phosphate or TMB as a substrate. Absorbance at 405 nm (with a reference at 540 nm) was measured using a Bio-Rad Model 550 Microplate Reader, as described previously [24]. OVA-specific IgG was measured by ELISA using mouse anti-OVA IgG antibody assay kit (Chondrex).

### Statistical Analysis

The paired Student’s *t* test was used to compare paired groups. Analysis of variance (ANOVA) was used for multi-group analysis. *P* values<0.05 were considered significant.

## Results

### LPS-liposomes Induce IFN-β Production but not Inflammatory Cytokines in BMDCs

**Figure 1:**
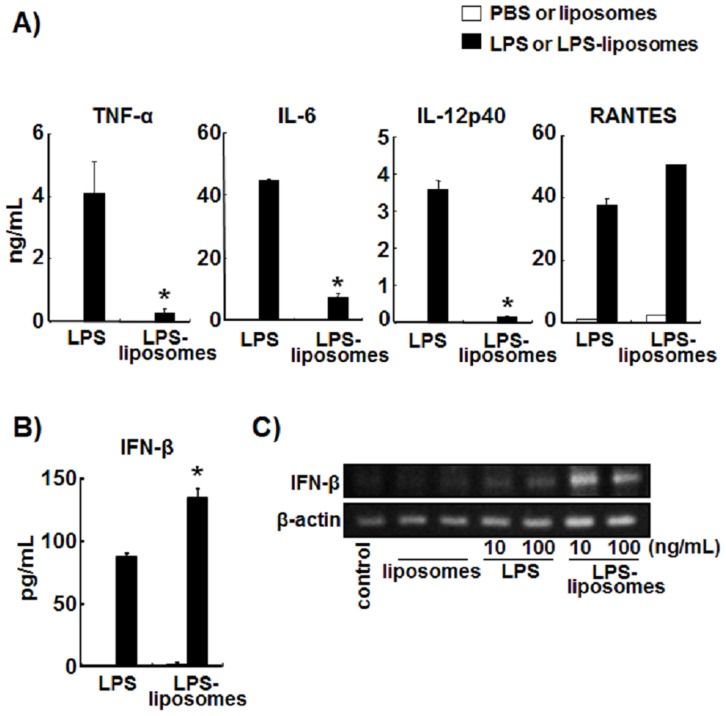
Lipopolysaccharide (LPS)-liposomes induce interferon (IFN)-β production but not inflammatory cytokines in bone marrow-derived dendritic cells (BMDCs). (A) BMDCs from wild-type (WT) mice (1.5×10^5^) were stimulated with LPS (100 ng/mL) or LPS-liposomes (100 ng/mL) for 2 h (TNF-α) or 24 h (IL-6, IL-12p40, and RANTES). Cytokine levels were determined by ELISA. (B) BMDCs from wild-type (WT) mice (1.5×10^5^) were stimulated with LPS (100 ng/mL) or LPS-liposomes (100 ng/mL) for 9 h. IFN-β levels were determined by ELISA. (C) BMDCs (1×10^6^) from WT mice were stimulated with LPS or LPS-liposomes (10 or 100 ng/mL) for 4 h and expression of IFN-β and β-actin mRNAs was determined by reverse transcription-polymerase chain reaction. PBS was control for LPS and liposomes was control for LPS-liposomes (Open columns). Data in A and B are average of three independent experiments. The values represent means ± S.E.M *P<0.05 (LPS *vs.* LPS-liposomes). Data shown in C is representative of three independent experiments.

First, we examined the production of cytokines and chemokines, which are induced via the MyD88-dependent and TRIF-dependent pathways. MyD88-dependent cytokines such as TNF-α, IL-6, and IL-12 were highly produced by BMDCs in response to LPS stimulation. By contrast, LPS-liposomes did not induce these inflammatory cytokines. The production of RANTES, a TRIF-dependent chemokine, was induced by both LPS and LPS-liposomes (Fig. 1A). Furthermore, the production of IFN-β, a TRIF-dependent cytokine, was significantly up-regulated by LPS-liposome stimulation compared with LPS (Fig. 1B), and the expression of IFN-β mRNA was also highly induced by LPS-liposome stimulation (Fig. 1C). Additionally, the production of IL-12 was completely inhibited in MyD88^−/−^ BMDCs by LPS, and the production of IFN-β was significantly inhibited in TRIF^−/−^ BMDCs by LPS and LPS-liposomes (Fig. S1). These results suggest that LPS-liposomes activate only the TRIF-dependent pathway.

### LPS-liposomes Induce IRF-3 Activation but not NFκB Activation in the Early Phase

**Figure 2:**
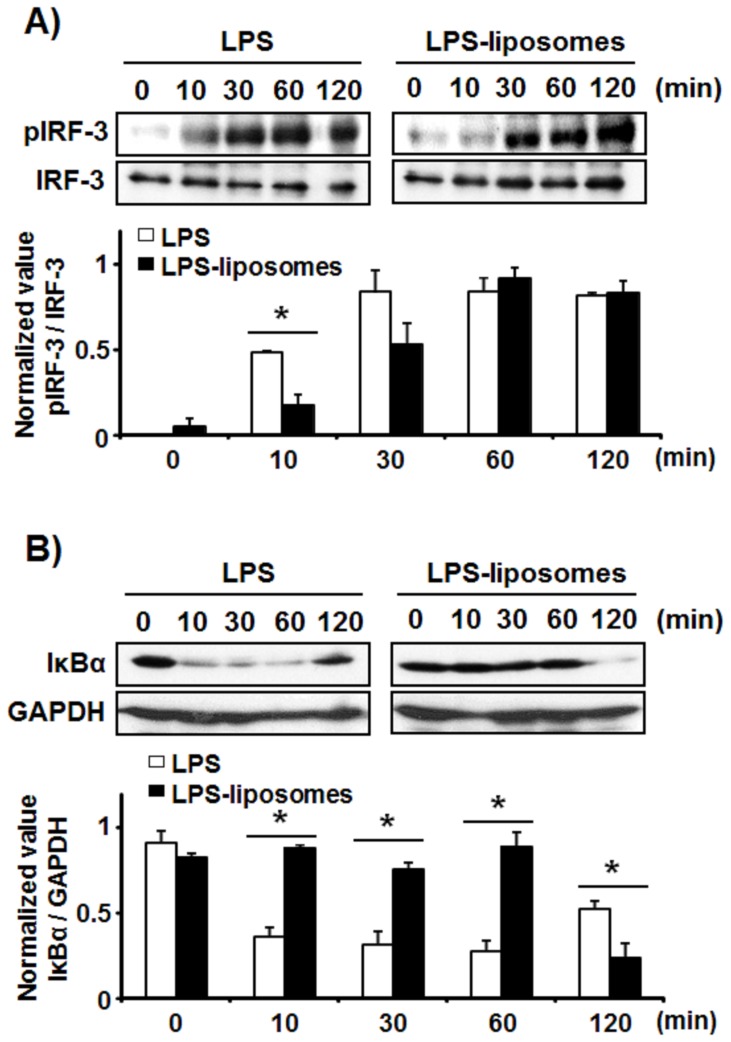
LPS-liposomes induce IRF-3 activation but not NFκB activation in the early phase. BMDCs (1×10^6^) from WT mice were stimulated with LPS (100 ng/mL) or LPS-liposomes (100 ng/mL) for 0–120 min. The cells were then lysed and the extracts were immunoblotted with the indicated antibodies. Data are average of three independent experiments and band intensity was quantified with Image J 1.45. The values represent means ± S.E.M *P<0.05.

It has been reported that the activation of NFκB in the early phase is induced by MyD88-dependent signaling, and the activation of IRF-3 and NFκB in the late phase is induced by TRIF-dependent signaling [9]. We next examined IRF-3 activation by detecting its phosphorylation, and NFκB activation by detecting the degradation of IκBα which inhibits NFκB activation. As shown in Figure 2A, phosphorylation of IRF-3 was induced by both LPS and LPS-liposomes in BMDCs. In the case of IκBα, early phase degradation was significantly detected in response to LPS, and late phase degradation was significantly stronger in response to LPS-liposome stimulation (Fig. 2B). These results also support that LPS-liposomes activate only the TRIF-dependent pathway.

### LPS-liposomes Induce Co-stimulatory Molecules in DCs as Efficiently as LPS without Inducing Excessive Production of TNF-α

**Figure 3:**
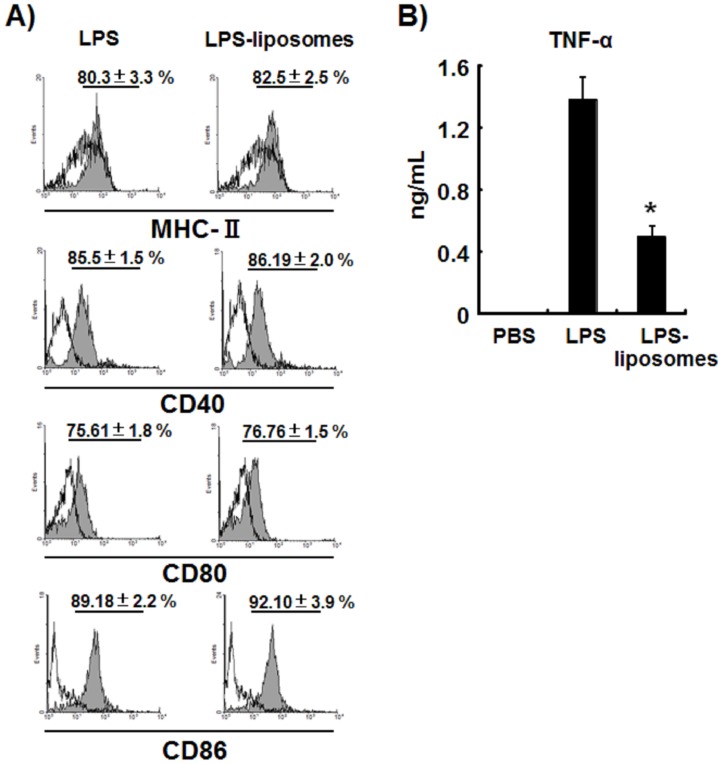
LPS-liposomes induce co-stimulatory molecules in DCs as efficiently as LPS without inducing excessive production of TNF-α. (A) WT mice were intravenously treated with LPS (10 µg per mouse) or LPS-liposomes (10 µg per mouse). At 6 h after treatment, splenocytes were harvested and the expression of MHC-II, CD40, CD80, and CD86 on CD11c^+^ splenic DCs was analyzed by flow cytometry. Splenic DCs from PBS treated mice were overlaid as control (open histograms). Percentage (%) are average of three independent experiments. *n* = 3 animals per group. The values represent means ± S.E.M *P<0.05. (B) WT mice were intravenously treated with LPS or LPS-liposomes (10 µg per mouse). At 1.5 h after treatment, sera were harvested and the level of TNF-α in the sera was determined by ELISA. Data are averages of three independent experiments. *n* = 5 animals per group. The values represent means ± S.E.M *P<0.05 (LPS *vs.* LPS-liposomes).

MHC-class II and co-stimulatory molecules such as CD40, CD80, and CD86 are important to stimulate naïve T cells to differentiate into effector T helper cells. LPS has been shown to induces the expression of co-stimulatory molecules in MyD88^−/−^ DCs, suggesting that the TRIF-dependent pathway plays a critical role in the induction of adaptive immunity [25]. In fact, TRIF^−/−^BMDCs did not induce the expression of CD40 and CD86 by LPS and LPS-liposomes compared with MyD88^−/−^BMDCs (Fig. S2). Therefore, we next examined the expression of co-stimulatory molecules in response to LPS and LPS-liposomes *in vivo*. WT mice were intravenously treated with LPS or LPS-liposomes, and splenic DCs were analyzed using a flow cytometer. As expected, LPS-liposomes induced the expression of MHC-class II, CD40, CD80, and CD86 as efficiently as LPS (Fig. 3A). Furthermore, the treatment of LPS-liposomes did not induce the excessive production of TNF-α *in vivo*, compared with LPS (Fig. 3B).

### LPS-liposomes Enhance Th1-mediated Antibody Responses

**Figure 4:**
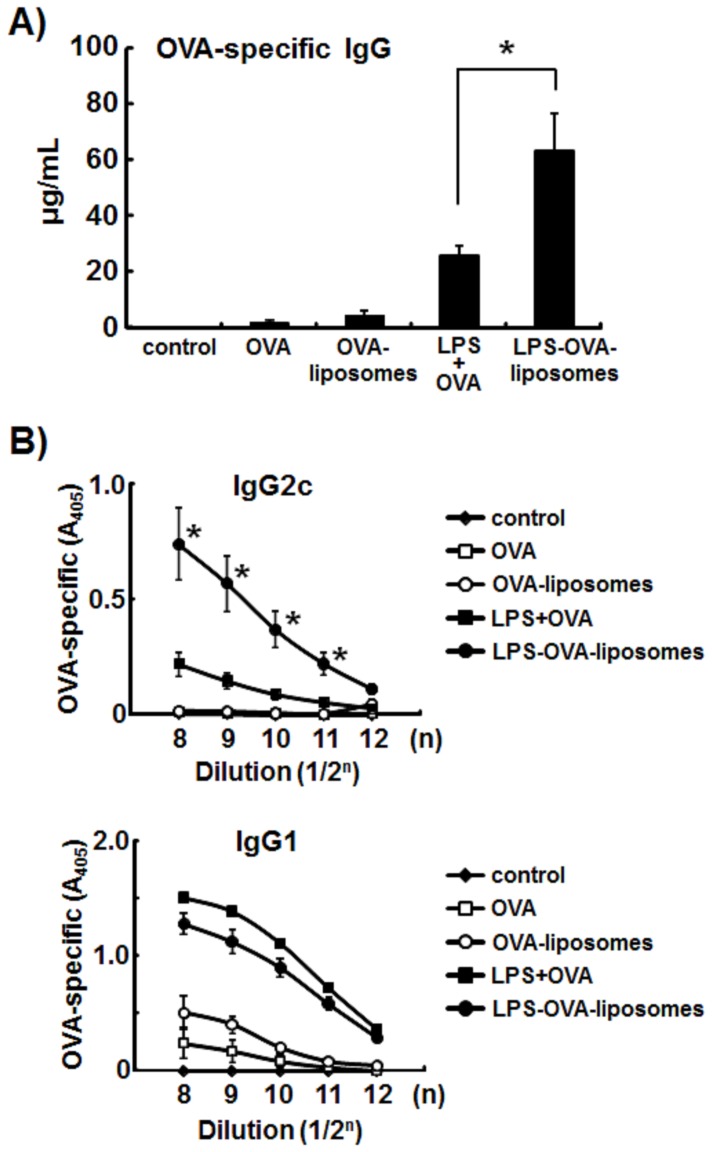
LPS-liposomes enhance Th1- mediated antibody responses. WT mice were immunized with LPS+OVA, OVA-liposomes, or LPS-OVA-liposomes (10 µg each per mouse, i.v.) and boosted after 2 weeks. After a further 2 weeks, sera were harvested and OVA-specific IgG, IgG1, and IgG2c levels were determined by ELISA. Data are averages of three independent experiments. *n* = 5 animals per group. The values represent means ± S.E.M *P<0.05.

Next, we prepared OVA-encapsulating LPS-liposomes (LPS-OVA-liposomes) to examine the induction of antigen-specific immune responses. WT mice were immunized with LPS plus OVA or LPS-OVA-liposomes and boosted after 2 weeks. After a further 2 weeks, sera were harvested and OVA-specific antibodies were examined by ELISA. As shown in Figure 4A, the production of OVA-specific total IgG was enhanced when LPS was added with OVA. LPS-OVA-liposomes elicited more effective production of OVA-specific total IgG. Surprisingly, LPS-OVA-liposomes significantly enhanced the production of OVA-specific IgG2c, which are a Th1-mediated immune response (Fig. 4B). On the other hand, the production of OVA-specific IgG1, which is a Th2-mediated immune response, was slightly decreased in response to LPS-OVA-liposome treatment compared with the OVA plus LPS treatment (Fig. 4B).

### Critical Role of Type-I IFN in LPS-liposome–induced Th1-immune Responses

**Figure 5:**
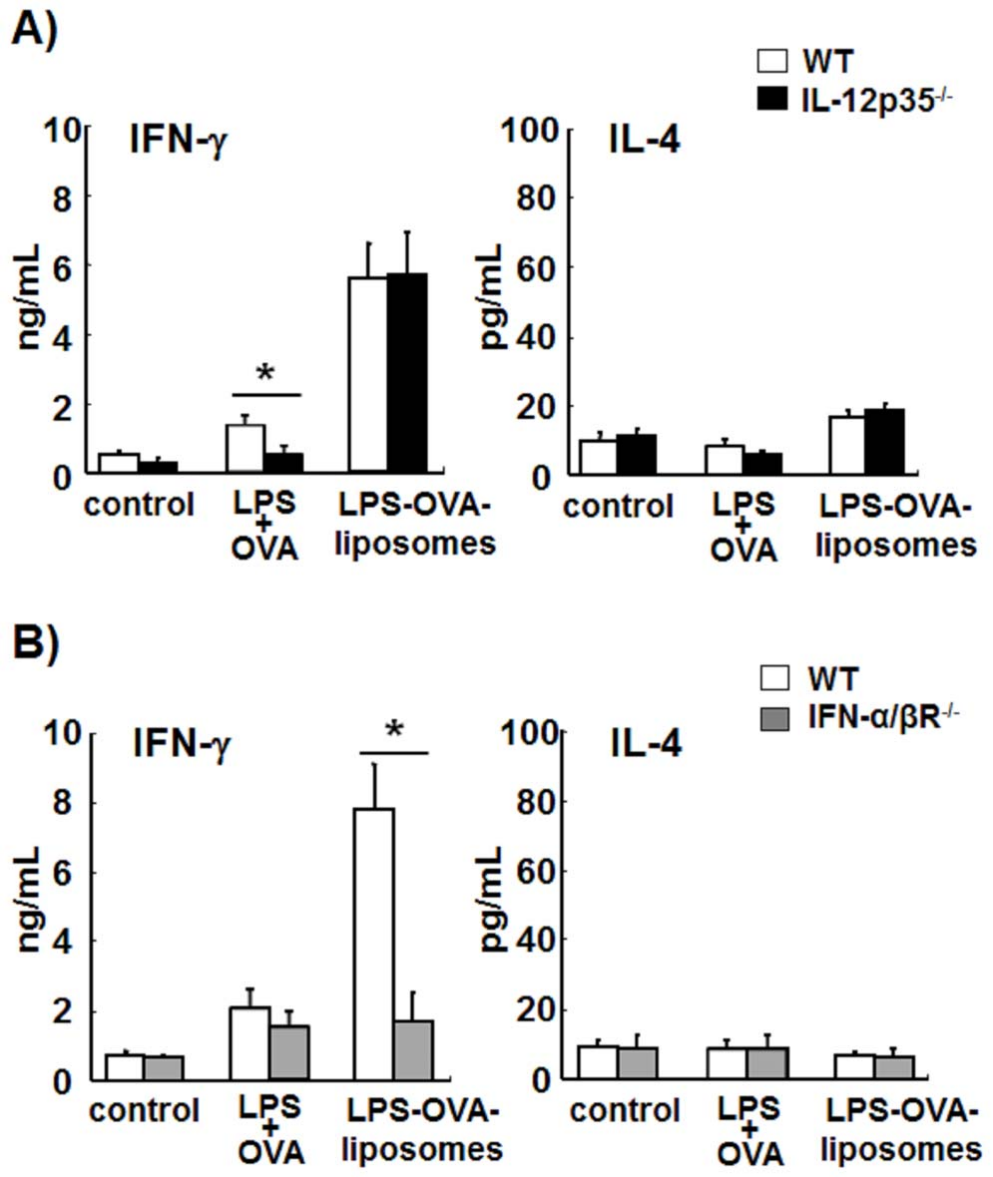
Critical role of type-I IFN in LPS-liposome–induced Th1-immune responses. WT, IL-12p35^−/−^ (A), and IFN-α/βR^−/−^ mice (B) were immunized with LPS plus OVA or LPS-OVA-liposomes (10 µg each per mouse, i.v.). After 1 week, the spleens were removed and the splenocytes (5×10^5^) were incubated with OVA (1 mg/mL) for 72 h, and the levels of IFN-γ and IL-4 were determined by ELISA. Data are averages of three independent experiments. *n* = 3 animals per group. The values represent means ± S.E.M *P<0.05.

LPS-liposomes activate only the TRIF-dependent pathway but not the MyD88-dependent pathway. Indeed, LPS-liposomes induced type-I IFN but not IL-12 (Fig. 1A). It is well established that IL-12 is important for the differentiation of naïve T cells into Th1 cells [13]. Therefore, we next examined the induction of antigen-specific Th1-immune response by LPS plus OVA and LPS-OVA-liposomes in IL-12^−/−^ mice. Mice were immunized with LPS plus OVA or LPS-OVA-liposomes. After 1 week, splenocytes from immunized mice were incubated with OVA for 72 h and IFN-γ and IL-4 levels were determined by ELISA. The production of IFN-γ, a Th1-mediated immune response, and the production of IL-4, a Th2-mediated immune response, by LPS-OVA-liposomes were not altered in IL-12p35^−/−^ mice compared with WT mice (Fig. 5A). On the other hand, the production of IFN-γ by LPS plus OVA was significantly decreased in IL-12p35^−/−^ mice (Fig. 5A). These results suggest that LPS-OVA-liposomes induce antigen-specific Th1-immune responses independent of IL-12. Therefore, we next focused on type-I IFN, which is induced by LPS-liposomes via the TRIF-dependent pathway. As shown in Figure 5B, IFN-γ production was completely inhibited in IFN-α/βR^−/−^ mice, strongly suggesting that the Th1-immune responses induced by LPS-liposomes depend on the induction of type-I IFN.

### Antigen-specific IgG2c Production by LPS-liposomes Depends on Type-I IFN

**Figure 6:**
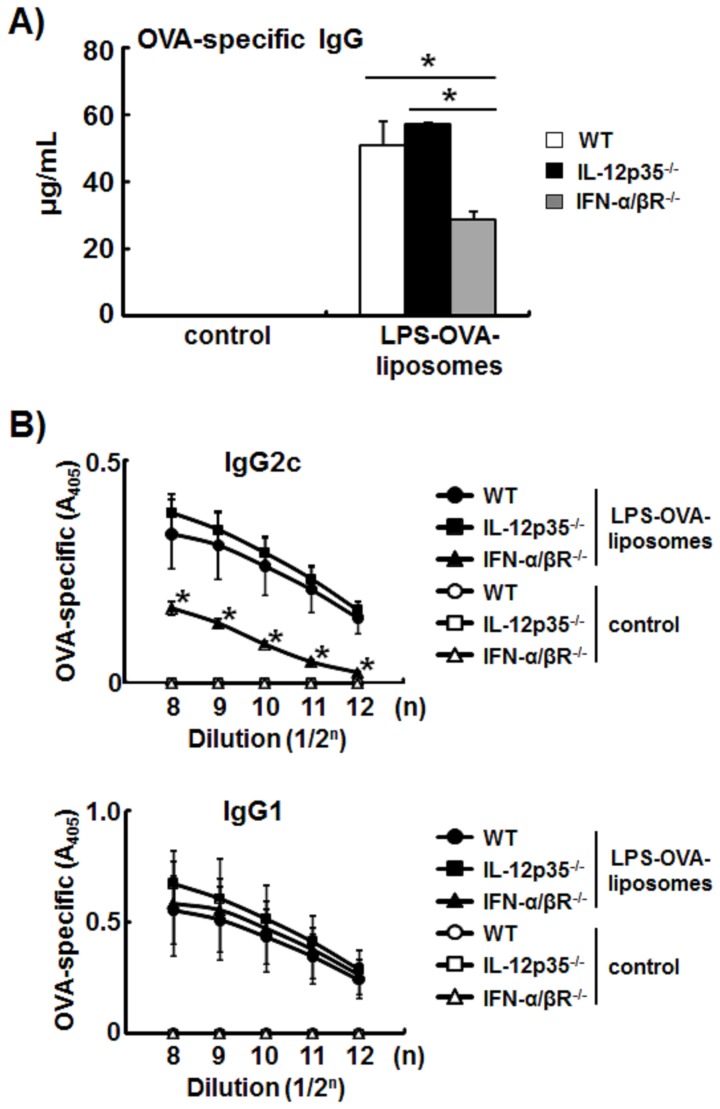
Antigen-specific IgG2c production by LPS-liposomes depends on type-I IFN. WT, IL-12p35^−/−^, and IFN-α/βR^−/−^ mice were immunized with LPS plus OVA or LPS-OVA-liposomes (10 µg each per mouse, i.v.), and boosted after 2 weeks. After a further 2 weeks, sera were harvested and OVA-specific IgG, IgG1, and IgG2c levels were determined by ELISA. Data are averages of three independent experiments. *n* = 5 animals per group. The values represent means ± S.E.M *P<0.05.

Finally, we examined the production of OVA-specific IgG2c, a Th1-mediated immune response, by LPS-OVA-liposomes in IL-12p35^−/−^ and IFN-α/βR^−/−^ mice. The production of OVA-specific total IgG was significantly decreased in IFN-α/βR^−/−^ mice (Fig. 6A). As expected, the production of OVA-specific IgG2c was also significantly decreased in IFN-α/βR^−/−^ mice but not in IL-12p35^−/−^ mice, whereas the production of OVA-specific IgG1 showed any change (Fig. 6B). These results also support that the Th1-immune responses induced by LPS-liposomes depend on type-I IFN but not IL-12.

## Discussion

To develop effective and safe immune adjuvants, it is important to generate strong and long immune responses without inducing unnecessary inflammation. Aluminum hydroxide is currently the major human vaccine adjuvant approved. Although it is effective in generating antibody responses, it requires repeated administration and tends to generate anti-parasitic Th2-immune responses, rather than anti-viral and anti-bacterial Th1-immune responses [26]. Recently, TLR ligands have been considered as candidate for immune adjuvants, given their ability to induce strong Th1-immune responses. However, these strong immune responses also induce unnecessary inflammation, which are sometimes fatal. Thus, it is very difficult to develop effective and safe immune adjuvants.

LPS can induce strong Th1-immune responses, but also induces unnecessary inflammations, which are induced via the MyD88-dependent pathway. In the present study, we newly prepared LPS-liposomes to deliver LPS into endosomes via endocytosis [18]. LPS-liposomes initiate only TRIF-dependent signaling via clathrin-mediated endocytosis, and do not induce the production of TNF-α and IL-6 but induce RANTES production in peritoneal macrophages [18]. In fact, LPS-liposomes also initiate only TRIF-dependent signaling in DCs which is essential for inducing adaptive immune responses (Figs. 1, 2, S1, S2). It is reported that the TRIF-dependent pathway is important for the induction of adaptive immune responses [15], [16]. As expected, like LPS, LPS-liposomes induced the expression of MHC-class II and co-stimulatory molecules such as CD40, CD80, and CD86, mediated by the TRIF-dependent pathway (Fig. 3A). Additionally, the treatment of LPS-liposomes did not induce the excessive production of TNF-α *in vivo*, compared with LPS (Fig. 3B). These results strongly suggest that LPS-liposomes activate only the TRIF-dependent pathway without inducing unnecessary inflammations via the MyD88-dependent pathway. The activation of transcription factors further supports that LPS-liposomes are TRIF-biased immune adjuvants (Fig. 2).

A recent study reported that LPS derivatives, such as MPLA, can be used as vaccine adjuvants with low toxicity [17]. These LPS derivatives are called TRIF-biased TLR4 agonists, and immunization with these agonists tends to activate the TRIF-dependent pathway rather than the MyD88-dependent pathway. This study also supports our observations that TRIF-biased immune adjuvants could serve as safe immune adjuvants. MPLA is a safe adjuvant, however, its ability to induce immune responses is weaker than that of LPS [17]. It has also been reported that MPLA can induce Th1-immune responses, but IFN-γ treatment is necessary and IL-12 production from DCs was also important [27]. Hence, the new method of inducing Th1-immune responses, without using MyD88-dependent pathway is needed.

**Figure 7:**
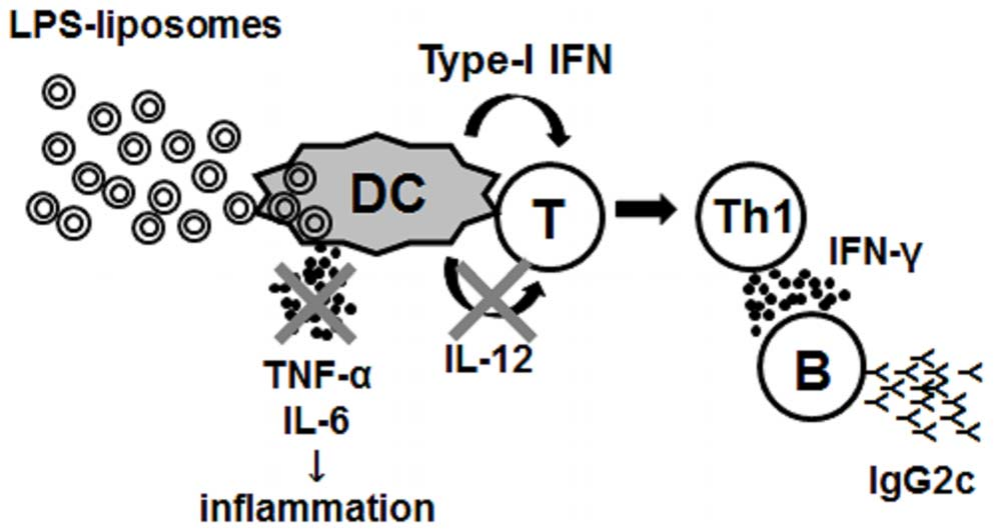
Model of the induction of Th1-immune responses by LPS-liposomes. Unlike cell surface LPS, intracellular delivery of LPS by liposomes does not induce inflammatory cytokine production, but induces type-I IFN. IL-12 was thought to be required for Th1-immunity induced by LPS. However, LPS-liposomes showed the critical role of type-I IFN in the induction of Th1-immunity.

LPS-liposomes induce effective antibody responses and Th1-mediated antibody responses compared with LPS (Fig. 4). Surprisingly, LPS-liposomes induced Th1-immune responses independent of IL-12 (Figs. 5, 6). We discovered that type-I IFN, but not IL-12, plays a critical role in the induction of Th1-immune responses by LPS-liposomes (Fig. 7). This new mechanism may aid the development of new vaccines against tumors and infections, given that type-I IFN elicits anti-tumor and anti-viral effects besides the induction of Th1-immune responses [28]–[30].

In conclusion, LPS-liposomes can effectively induce Th1-immune responses without inducing unnecessary inflammation, and may be useful as an immune adjuvant to induce protective immunity (Fig. 7).

## Supporting Information

Figure S1 **LPS and LPS-liposomes induce IFN-β production through the TRIF-dependent pathway in BMDCs.** MyD88^−/−^ and TRIF^−/−^ BMDCs (1.0×10^5^) were stimulated with LPS (100 ng/mL) or LPS-liposomes (100 ng/mL) for 9 h (IFN-β) or 24 h (IL-12p40). Cytokine levels were determined by ELISA. Data are average of two independent experiments. The values represent means ± S.E.M *P<0.05 (MyD88^−/−^ *vs.* TRIF^−/−^).
(TIF)

Figure S2 **LPS and LPS-liposomes induce co-stimulatory molecules through the TRIF-dependent pathway in BMDCs.** MyD88^−/−^ and TRIF^−/−^ BMDCs (5.0×10^5^) were stimulated with LPS or LPS-liposomes (100 ng/mL) for 48 h, and the expression of CD40 and CD86 on CD11c^+^ BMDCs were analyzed by flow cytometry. PBS treated BMDCs were overlaid as control (black lines). Percentage (%) are average of three independent experiments. The values represent means ± S.E.M *P<0.05 (*vs.* MyD88^−/−^).
(TIF)
