# Supplementary figures and images for: Intracellular Delivery of Lipopolysaccharide Induces Effective Th1-Immune Responses Independent of IL-12
Source: PLoS One. 2013 Jul 17;8(7):e68671. doi: 10.1371/journal.pone.0068671 (PMC3714268; doi:10.1371/journal.pone.0068671)

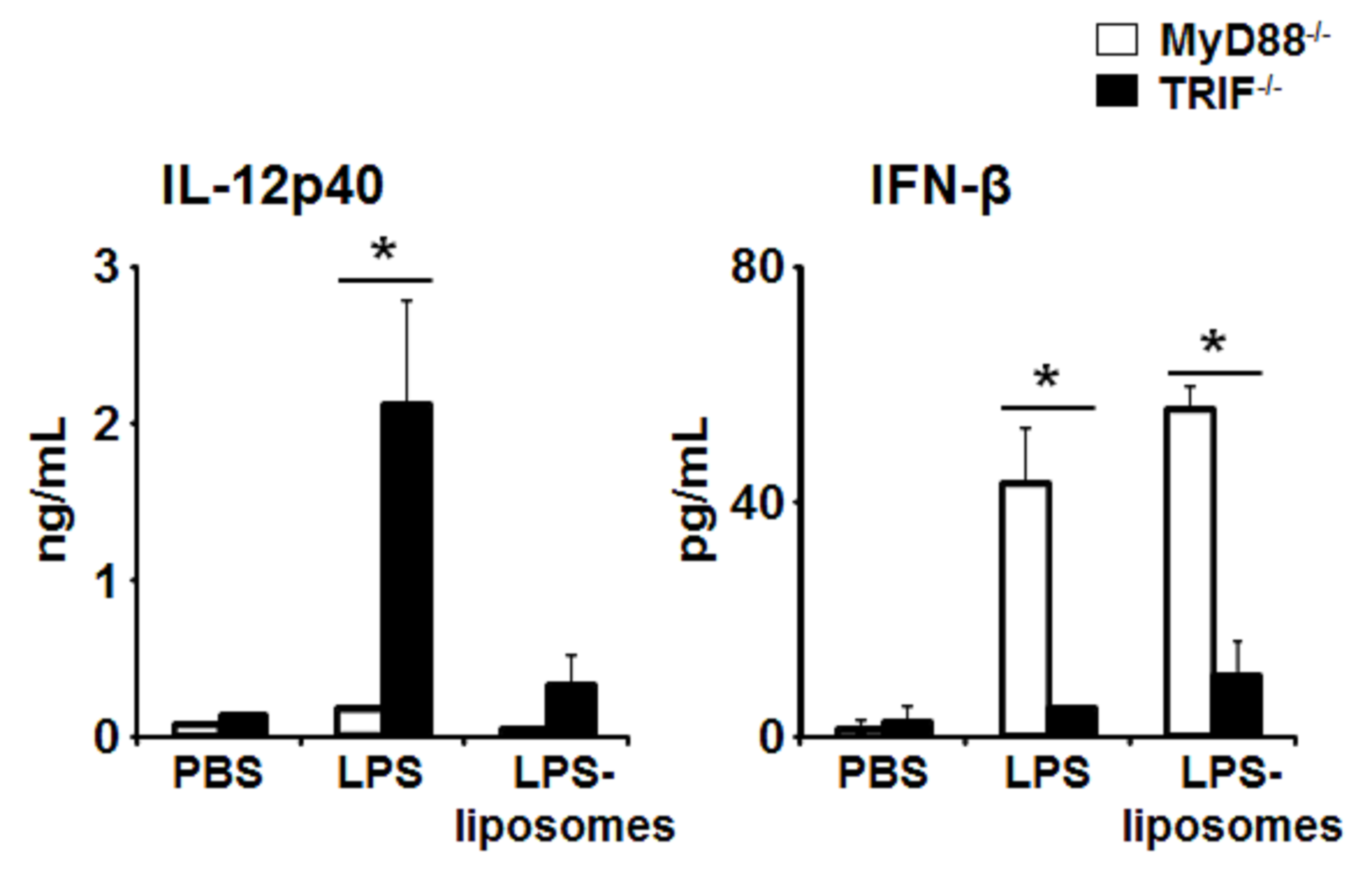

Supplement: Figure S1 — LPS and LPS-liposomes induce IFN-β production through the TRIF-dependent pathway in BMDCs. MyD88−/− and TRIF−/− BMDCs (1.0×10^5^) were stimulated with LPS (100 ng/mL) or LPS-liposomes (100 ng/mL) for 9 h (IFN-β) or 24 h (IL-12p40). Cytokine levels were determined by ELISA. Data are average of two independent experiments. The values represent means ± S.E.M *P<0.05 (MyD88−/− vs. TRIF−/−). (TIF) [file pone.0068671.s001.tif]

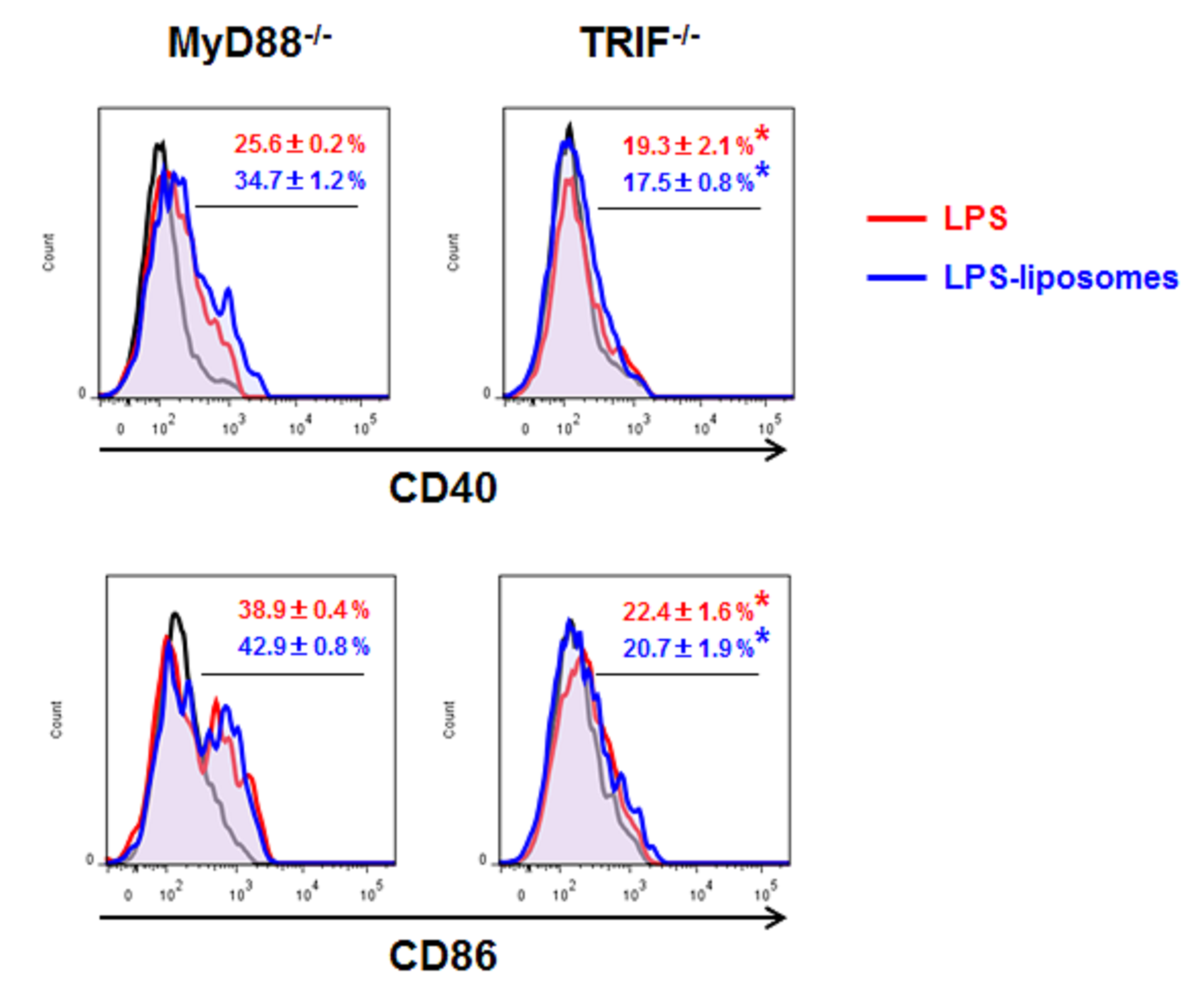

Supplement: Figure S2 — LPS and LPS-liposomes induce co-stimulatory molecules through the TRIF-dependent pathway in BMDCs. MyD88−/− and TRIF−/− BMDCs (5.0×10^5^) were stimulated with LPS or LPS-liposomes (100 ng/mL) for 48 h, and the expression of CD40 and CD86 on CD11c+ BMDCs were analyzed by flow cytometry. PBS treated BMDCs were overlaid as control (black lines). Percentage (%) are average of three independent experiments. The values represent means ± S.E.M *P<0.05 (vs. MyD88−/−). (TIF) [file pone.0068671.s002.tif]
